# Hypoxia regulates global membrane protein endocytosis through caveolin-1 in cancer cells

**DOI:** 10.1038/ncomms11371

**Published:** 2016-04-20

**Authors:** E. Bourseau-Guilmain, J. A. Menard, E. Lindqvist, V. Indira Chandran, H. C. Christianson, M. Cerezo Magaña, J. Lidfeldt, G. Marko-Varga, C. Welinder, M. Belting

**Affiliations:** 1 Department of Clinical Sciences, Section of Oncology and Pathology, Lund University, 221 85 Lund, Sweden; 2 Center of Excellence in Biological and Medical Mass Spectrometry (CEBMMS), Lund University, 221 84 Lund, Sweden; 3 First Department of Surgery, Tokyo Medical University, Tokyo 160-0023, Japan; 4 Clinical Protein Science & Imaging, Biomedical Center, Biomedical Engineering, Lund University, 221 84 Lund, Sweden; 5 Department of Oncology and Radiotherapy, Skåne University Hospital, 221 85 Lund, Sweden

## Abstract

Hypoxia promotes tumour aggressiveness and resistance of cancers to oncological treatment. The identification of cancer cell internalizing antigens for drug targeting to the hypoxic tumour niche remains a challenge of high clinical relevance. Here we show that hypoxia down-regulates the surface proteome at the global level and, more specifically, membrane proteome internalization. We find that hypoxic down-regulation of constitutive endocytosis is HIF-independent, and involves caveolin-1-mediated inhibition of dynamin-dependent, membrane raft endocytosis. Caveolin-1 overexpression inhibits protein internalization, suggesting a general negative regulatory role of caveolin-1 in endocytosis. In contrast to this global inhibitory effect, we identify several proteins that can override caveolin-1 negative regulation, exhibiting increased internalization at hypoxia. We demonstrate antibody-mediated cytotoxin delivery and killing specifically of hypoxic cells through one of these proteins, carbonic anhydrase IX. Our data reveal that caveolin-1 modulates cell-surface proteome turnover at hypoxia with potential implications for specific targeting of the hypoxic tumour microenvironment.

## Abstract (web summary)

Hypoxia promotes tumour aggressiveness and resistance of cancers to oncological treatment. Here, the authors show that caveolin-1 can down-regulate global membrane protein endocytosis in hypoxic cells with potential implications for targeting the hypoxic 3microenvironment of aggressive tumours.

## Graphical abstract

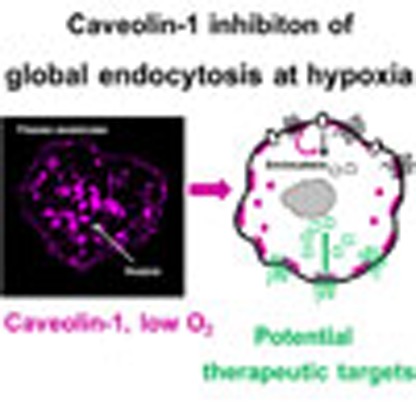

Cancer cells thrive within a complex milieu characterized by hypoxia that plays a fundamental role in tumour development^1^,^2^,^3^. Altogether, hypoxic stress-induced signalling select for tumour cells that will successfully adapt to their hostile microenvironment and drive disease progression by inducing, for example, angiogenesis, immune cell evasion, coagulation and cancer cell stemness. These responses further result in resistance to conventional cancer therapies, including radiotherapy and chemotherapy. An increased understanding of cancer cell adaptive mechanisms to hypoxia is critical for the development of improved strategies in the fight against cancer.

Abnormal trafficking of cell-surface receptors is involved in malignant transformation, and several endocytosis associated proteins are deregulated in cancer cells^4^. For example, overexpression of huntingtin-interacting protein 1, an adaptor for clathrin coat assembly, alters epithelial growth factor receptor (EGFR) trafficking during tumour development; mutant variants of hepatocyte growth factor receptor (HGFR) exhibit increased endocytosis, resulting in enhanced tumour progression; and ras protein (RAS)-induced macropinocytosis of platelet derived growth factor receptor beta can promote tumour progression^5^,^6^. Further, accumulating evidence indicates that cellular responses to the extracellular environment are regulated by the spatial coordination of cell-surface proteins and further uptake and sorting into vesicular compartments of the endocytic systems^4^. Interestingly, in some cases these mechanisms have been related to hypoxia, thereby contributing to an enhanced tumorigenic signalling^7^,^8^,^9^,^10^,^11^. Accordingly, cell-surface receptors with endocytic transport activity emerge as attractive targets for tumour-specific delivery of therapeutic substances, most importantly antibody-drug conjugates (ADCs) that are currently approved in the treatment of breast cancer and lymphoma^12^,^13^.

The overall effects of hypoxia on the cellular transcriptome, proteome and metabolome have been extensively studied, pointing at a diverse and relatively conserved response in malignant tumours of different origins. Here, we were interested in elucidating how hypoxia at a functional level regulates the plasma membrane proteome and its endocytic activity to better understand how to target the microenvironment of aggressive tumours. We have implemented a widely applicable method that integrates reversible membrane protein labelling with fluorescence-activated cell sorting (FACS), confocal microscopy imaging and quantitative proteomics analyses for the comprehensive visualization, quantification and identification of internalizing cell-surface proteins. Our data reveal that hypoxia modulates global cell-surface proteome endocytosis through caveolin-1 dependent mechanisms. These findings have potential implications for the spatial regulation of the receptor signalling pathways in tumour biology as well as for the development of treatment strategies specifically targeted at the tumour microenvironment.

## Results

### Hypoxia down-regulates global membrane proteome endocytosis

**Figure 1:**
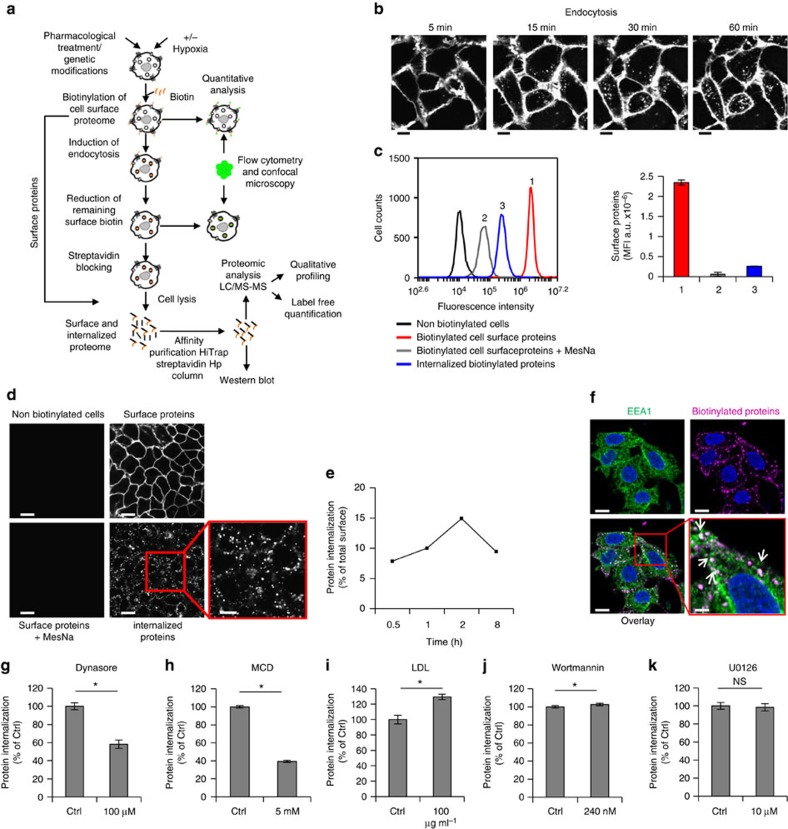
Dynamics of constitutive membrane protein endocytosis. (**a**) Schematic outline of procedures for the quantification and encoding of cell-surface proteome endocytosis, as described in Methods. (**b**) Representative confocal microscopy images of cell-surface and internalized biotinylated proteins in HeLa cells at the indicated time points (see also, Supplementary Movie 1). Scale bar, 20 μm. (**c**) FACS quantification of biotinylated cell-surface, and endocytosed membrane proteome in HeLa cells following 30 min of internalization; left panel: representative histograms of non-biotinylated cells, total cell-surface biotinylation, residual cell-surface signal following reductive cleavage of biotin-protein linker with MesNa and internalized membrane proteins; right panel: quantitative analysis presented as the mean±s.d. from three independent experiments each performed in duplicates. MFI, median fluorescence intensity. (**d**) Representative confocal microscopy images of HeLa cells from experiment described in (**c**). Scale bar, 20 μm; lower right panel, 10 μm. (**e**) Time-dependent internalization in HeLa cells of cell-surface proteome presented as % of total cell-surface biotinylation at *t*=0, that is, without induction of endocytosis. (**f**) Confocal microscopy imaging of biotinylated proteins (magenta) and the early endosome marker EEA1 (green) in HeLa cells shows co-localization following 30 min of endocytosis. Arrows in lower right panel indicate co-localization. Shown are representative images from at least 3 independent experiments. Scale bar, 10 μm; lower right panel, 3.5 μm. (**g–k**) FACS quantification of constitutive biotinylated membrane protein endocytosis at 30 min following pre-treatment of HeLa cells with (**g**) dynamin inhibitor Dynasore for 30 min, (**h**) membrane cholesterol depletion agent methyl-β-cyclodextrin (MCD) for 30 min, (**i**) low-density lipoprotein cholesterol loading for 20 h, (**j**) macropinocytosis inhibitor wortmannin for 1 h and (**k**) ERK1/2 phosphorylation inhibitor UO126 for 1 h at the indicated concentrations. Data are presented as % of untreated control cells (Ctrl)±s.d. from a representative experiment. NS, not significant. *P<0.05 (Student's *t*-test).

To comprehensively study the dynamics of cell-surface proteome internalization at various treatment conditions, we initially established optimized procedures for reversible protein labelling on HeLa cells using a cell membrane impermeable and cleavable biotinylation agent integrated with FACS, confocal microscopy imaging and quantitative proteomics analyses (Fig. 1a). Cell-surface proteome internalization was visualized by live cell confocal microscopy imaging, showing clearly visible translocation of cell-surface protein biotinylation to intracellular vesicles already at 5 min that continuously increased during 60 min (Supplementary Movie 1; representative images are shown in Fig. 1b). FACS experiments were performed to quantitatively investigate the fraction of the biotinylated membrane proteome that is endocytosed at various conditions. Importantly, treatment with the membrane impermeable reducing agent MesNa under optimized conditions reduced the residual cell-surface biotinylation signal to only ∼2% of the total signal (Fig. 1c). Moreover, the residual surface signal was virtually undetectable by confocal microscopy, while there was a significant intracellular signal from internalized, biotinylated proteins both by FACS and confocal imaging (Fig. 1c,d). Using this approach, we could demonstrate a time-dependent increase of membrane protein internalization; at 2 h ∼15% of the total cell-surface proteome was internalized, and then showed a decline at 8 h to ∼10% probably due to protein degradation (Fig. 1e). To further corroborate that the biotinylation signal was intracellular and associated with an endocytic process, cells were co-stained for biotin and EEA1, showing a clear co-association by confocal microscopy (Fig. 1f). Moreover, we pre-treated cells with established inhibitors of the major endocytic pathways including dynamin-dependent (Supplementary Fig. 1a) and membrane raft-dependent endocytosis (Supplementary Fig. 1b). Constitutive protein internalization was significantly perturbed on treatment with the small molecule dynamin inhibitor dynasore^14^ as well as by disruption of cholesterol-rich membrane domains using methyl-β-cyclodextrin (MCD) (Fig. 1g,h). Accordingly, cellular cholesterol pre-loading with low-density lipoprotein resulted in significantly increased constitutive protein internalization (Fig. 1i). However, we found no major role of PI3K-dependent macropinocytosis (Fig. 1j and Supplementary Fig. 1c) or intact ERK1/2 signalling (Fig. 1k and Supplementary Fig. 1d) in constitutive internalization under these conditions.

**Figure 2:**
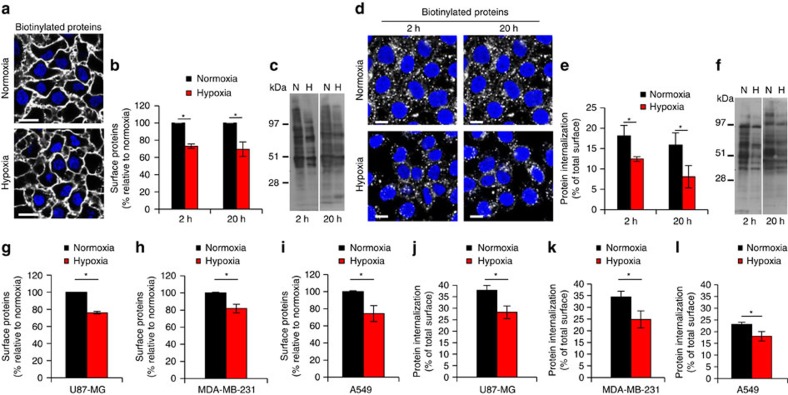
Hypoxia down-regulates global membrane protein endocytosis. (**a**) HeLa cells were pre-treated at normoxia or hypoxia (1% O_2_) for 20 h, followed by cell-surface biotinylation, staining with streptavidin-AF-488 and visualization by confocal microscopy. Scale bar, 20 μm. (**b**) FACS quantification of biotinylated cell-surface proteome in HeLa cells shows inhibition by hypoxic treatment for the indicated time periods. (**c**) Immunoblotting for biotinylated cell-surface proteins from a similar experiment as described in (**b**) shows down-regulation by hypoxia. (**d**) Confocal microscopy imaging of endocytosed, biotinylated membrane proteins following 30 min of internalization and cell-surface biotinylation depletion in HeLa cells shows inhibition by hypoxic treatment for the indicated time periods. Scale bar, 10 μm. (**e**) FACS quantification of the endocytosed membrane proteome from a similar experiment as described in (**d**) shows down-regulation by hypoxia. (**f**) Immunoblotting for endocytosed proteins from a similar experiment as described in (**d**) shows inhibition by hypoxia. (**g–l**) FACS quantification of biotinylated cell-surface proteome (**g**–**i**) and endocytosed membrane proteome (**j**–**l**) following 30 min of internalization performed with the indicated cell types (A549, lung adenocarcinoma; MDA-MB-231, breast adenocarcinoma; U87-MG, glioblastoma) treated at normoxia or hypoxia for 20 h. Data are presented as % relative to normoxic cells (in **b** and **g–i**) or as % of total cell-surface biotinylation at normoxia and hypoxia, respectively, at *t*=0 (in **e**, and **j–l**)±s.d. from three independent experiments. *P<0.05 (Student's *t*-test).

With the above data, we were able to address fundamental questions related to the global effect of hypoxic stress on cell-surface protein expression and, more importantly, how hypoxia may regulate the functional activity of endocytic membrane proteins. Initially, cells were subjected to normoxia or prolonged hypoxia (20 h), and then surface biotinylated and kept at 4 °C to avoid endocytosis, followed by visualization of the biotinylated cell-surface proteome. Confocal microscopy imaging suggested a moderate reduction of surface fluorescence intensity in hypoxic as compared with normoxic cells (Fig. 2a). To explore this further, we next performed quantitative studies by FACS and found that already in the acute phase of hypoxia (2 h), the cell-surface proteome was significantly down-regulated by ∼27% as compared with normoxic control cells, an effect that remained at prolonged hypoxia (Fig. 2b). These results, which probably reflect the known reduction of protein translation by hypoxic stress^15^ were further supported by immunoblotting experiments for biotinylated cell-surface proteins, showing a generally weaker signal in hypoxia at both time points (Fig. 2c; see Supplementary Fig. 1e for protein loading control). In the next series of experiments, normoxic or hypoxic cells were allowed to undergo endocytosis for 30 min after surface biotinylation, and the internalized fraction of the cell-surface proteome was visualized by confocal microscopy, showing an apparent decrease in acute as well as prolonged hypoxia (Fig. 2d). Interestingly, we found that hypoxic as compared with normoxic conditions resulted in an ∼50% reduction of the fraction of the surface proteome undergoing endocytosis, as quantified by FACS (Fig. 2e). Accordingly, immunoblotting experiments for endocytosed membrane proteins showed a clearly decreased signal in hypoxic cells both in the acute and prolonged settings (Fig. 2f; see Supplementary Fig. 1f for protein loading control). We conclude that hypoxia has a limited inhibitory effect on the total surface proteome and, importantly, a more pronounced inhibitory effect on the fraction of internalized membrane proteins.

Our findings may reflect a general inhibitory effect of hypoxia on the endocytic machinery; we thus determined the activity of the major endocytic pathways, including clathrin-dependent uptake of transferrin, macropinocytic dextran uptake and membrane raft-dependent uptake of Cholera Toxin subunit-B (binds strongly to GM1, monosialotetrahexosylganglioside) uptake in normoxic and hypoxic cells. At odds with this idea, we found a significant increase of both transferrin and cholera toxin uptake at hypoxic as compared with normoxic conditions, whereas dextran uptake was not significantly affected by hypoxia (Supplementary Fig. 2). We conclude that hypoxic stress reduced the overall membrane protein internalizing capacity through mechanisms that apparently were not associated with a general down-regulation of the major endocytic pathways. Importantly, this finding was not restricted to HeLa cells, as comparable results were obtained with additional cancer cell-lines from human glioblastoma multiforme (U87-MG), breast cancer (MDA-MB-231) and lung cancer (A549) (Fig. 2g–l).

### Hypoxic down-regulation of endocytosis depends on caveolin-1

**Figure 3:**
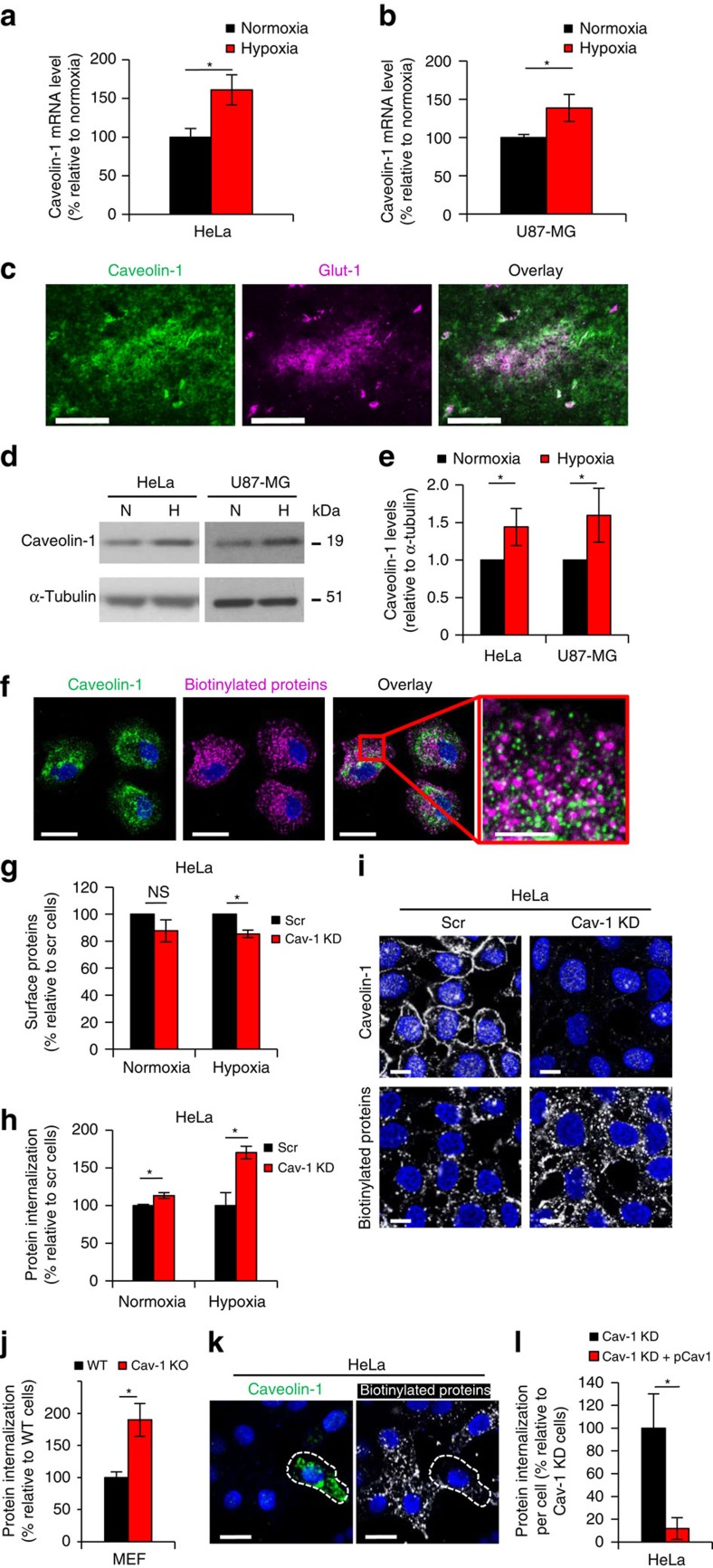
Caveolin-1 regulates constitutive membrane protein endocytosis in hypoxic cells. Caveolin-1 mRNA levels in HeLa (**a**) and U87-MG (**b**) cells at hypoxia or normoxia presented as % relative to normoxia±s.d. (*n*=3). (**c**) Immunofluorescence staining of a human glioblastoma tumour shows increased caveolin-1 in Glut-1-positive, hypoxic regions. Scale bar, 500 μm. (**d**) HeLa and U87-MG cell lysates from normoxic and hypoxic conditions were analysed for caveolin-1 by western blotting with tubulin as loading control. (**e**) Quantification of caveolin-1 to tubulin ratio in hypoxic versus normoxic cells (set to 1). Data represent the average±s.d. (*n*=3) (**f**) HeLa cells were surface biotinylated, followed by endocytosis for 30 min. Cells were stained for internalized proteins (magenta) and caveolin-1 (green). The indicated area shows weak co-localization. Scale bar, 20 μm; right panel, 5 μm. (**g**) FACS quantification of biotinylated cell-surface proteome in caveolin-1 knockdown (Cav-1 KD) and control HeLa cells transduced with a scrambled shRNA sequence (Scr) at normoxic and hypoxic conditions. (**h**) FACS quantification of the endocytosed proteome at 30 min from a similar experiment as in (**g**). Data are presented as % relative to Scr cells±s.d. (*n*=3). (**i**) Hypoxic Scr and Cav-1 KD HeLa cells were surface biotinylated, followed by endocytosis for 30 min. Cells were stained for internalized proteins and caveolin-1. Scale bar, 10 μm. (**j**) FACS quantification of the endocytosed proteome at 30 min in hypoxic WT and caveolin-1 knockout (Cav-1 KO) MEF cells. Data are presented as % relative to WT cells±s.d. (*n*=3). (**k**) Hypoxic Cav-1 KD cells transiently transfected with caveolin-1-expressing plasmid (pCav-1) were surface biotinylated followed by 30 min of endocytosis. Cells were stained for internalized proteins and caveolin-1, showing decreased internalization in caveolin-1 overexpressing cells (white dashed line). Scale bar, 20 μm. (**l**) Quantitative results from experiment described in (**k**) using Cell Profiler. Data are presented as % relative to nontransfected Cav-1 KD cells±s.d. from six representative areas (*n*=3). (**c**,**f**,**i**): Representative images from three independent experiments. *P<0.05 (Student's *t*-test).

To elucidate the mechanisms of hypoxic effects on membrane protein internalization, we performed gene array studies with cells from normoxic and hypoxic conditions, specifically focusing on proteins that may be involved in the regulation of membrane protein trafficking. In both HeLa (Fig. 3a) and U87-MG (Fig. 3b) cells, we found significant hypoxic induction of caveolin-1 mRNA. This was of interest as caveolin-1 has previously been implicated in tumour development, and as a major regulator of endocytosis^16^,^17^,^18^,^19^,^20^. Importantly, loss of caveolin-1 may increase the mobility of membrane raft components, and caveolin-1 has been shown to negatively regulate the endocytosis of, for example, autocrine motility factor, toxins, pathogens as well as exosomes^21^,^22^,^23^,^24^,^25^. We found significantly enhanced expression of caveolin-1 protein in hypoxic regions of glioblastoma patient tumours (Fig. 3c), as determined by co-staining for the well-established hypoxia marker glucose transporter-1 (Glut-1)^26^ as well as increased levels of caveolin-1 in hypoxic as compared with normoxic HeLa and U87-MG cells *in vitro*, as shown by immunoblotting (Fig. 3d,e). However, there was only a weak co-association between internalized membrane proteins and caveolin-1 (Fig. 3f), indicating that caveolin-1 positive endocytic vesicles are not directly involved or have only a minor role in constitutive protein internalization. This did not, however, exclude that caveolin-1 may stabilize and negatively regulate membrane protein internalization as caveolin-1 can inhibit endocytosis through an indirect mechanism that is not necessarily caveolae-associated^17^,^27^. To investigate this possibility, stable caveolin-1 knock-down HeLa cells were generated by lentiviral transduction, showing maintained reduction of caveolin-1 also at hypoxic conditions (Supplementary Fig. 3a). In normoxic cells, caveolin-1 deficiency did not significantly alter the total biotinylated surface proteome as compared with control cells stably transduced with a scrambled shRNA sequence (Fig. 3g). At hypoxic conditions, there was a significant but limited reduction (∼15%) of surface proteins in caveolin-1 deficient as compared with control cells (Fig. 3g). We found that caveolin-1 deficiency significantly increased surface protein internalization in normoxic cells (by ∼10%). Interestingly, this effect was substantially greater at hypoxic conditions with an ∼75% increase as compared with hypoxic control cells (Fig. 3h). Altogether, these results suggested that loss of caveolin-1 could reverse the inhibitory effect of hypoxia on global surface proteome internalization, and that this effect was not due to an overall increase of protein sorting to the cell-surface. This conclusion was supported by imaging studies, showing clearly enhanced protein internalization in caveolin-1 deficient as compared with control cells at hypoxic conditions (Fig. 3i). Moreover, we found that global protein internalization was increased by almost two fold at hypoxia (Fig. 3j and Supplementary Fig. 3b) while slightly decreased at normoxia (Supplementary Fig. 3c) in caveolin-1 knockout (KO) as compared with wild-type (WT) mouse embryonic fibroblast (MEF) cells, showing that loss of caveolin-1 increases protein internalization preferentially at hypoxia also in a well-defined system of caveolin-1 deficiency. To investigate whether this effect could be reversed, caveolin-1 was reintroduced into hypoxic caveolin-1 knockdown (KD) HeLa as well as MEF caveolin-1 KO cells by transfection with a caveolin-1 encoding plasmid. The results revealed that caveolin-1 overexpression dramatically inhibits global protein internalization (Fig. 3k,l and Supplementary Fig. 3d). Altogether, these findings provide evidence that caveolin-1 has a significant role as a negative regulator of surface protein internalization at conditions of hypoxic stress.

### Caveolin-1 inhibits dynamin-mediated endocytosis at hypoxia

**Figure 4:**
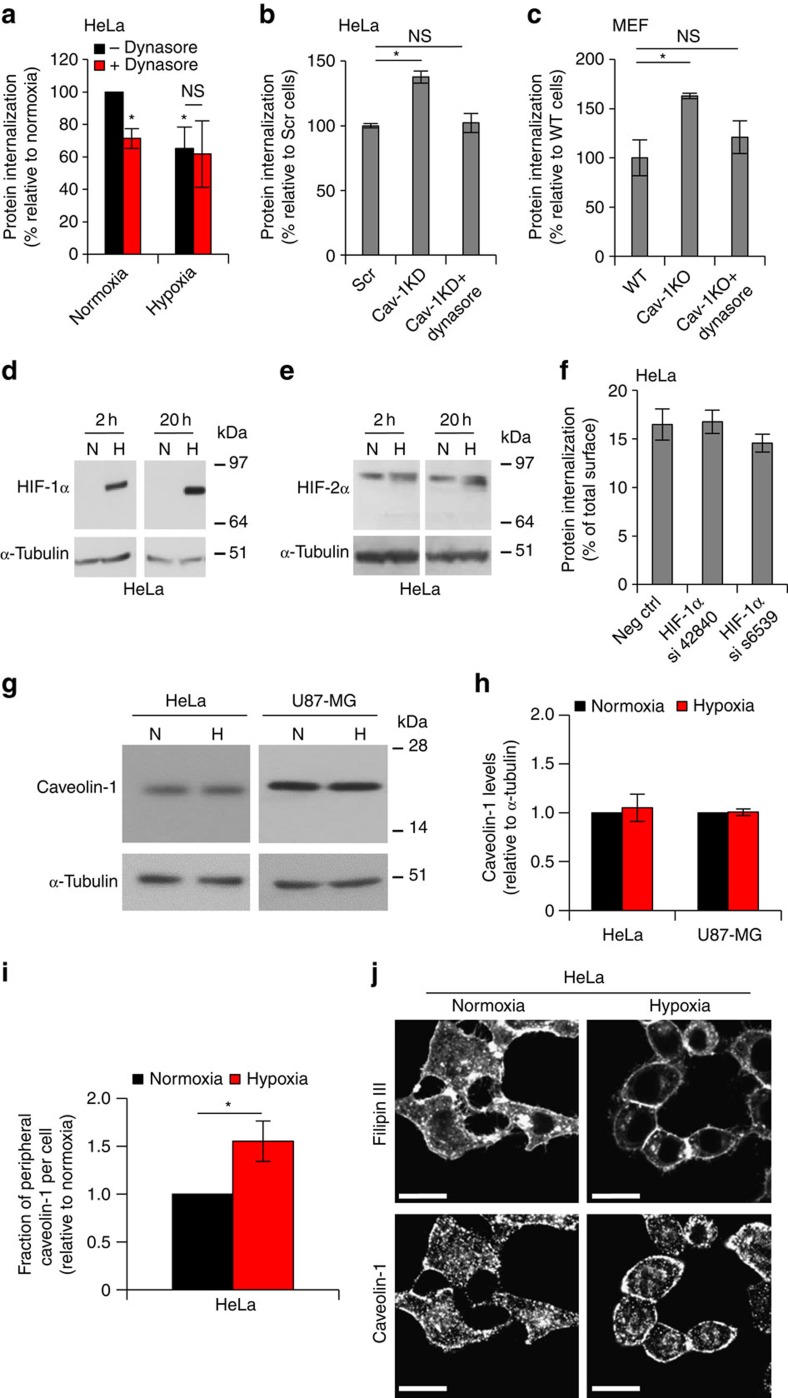
Caveolin-1 regulates dynamin-dependent endocytosis and is distributed to the cell periphery in hypoxia. (**a**) Dynamin inhibition mimics hypoxia. FACS quantification of constitutive biotinylated membrane protein endocytosis at 30 min in normoxic and hypoxic HeLa cells following no treatment (–Dynasore) or pre-treatment with dynamin inhibitor Dynasore (+Dynasore) for 30 min. (**b**) FACS quantification of the endocytosed proteome in hypoxic control (Scr) and caveolin-1 knockdown (Cav-1 KD) HeLa cells with and without Dynasore pre-treatment shows that dynamin inhibition reverses the effect of caveolin-1 deficiency. (**c**) Similar experiment as in (**b**) with WT and caveolin-1 knockout (Cav-1 KO) MEF cells. (**a–c**) Data are presented as % relative to respective controls±s.d. (*n*=3). *P<0.05 (Student's *t*-test). (**d**) HIF-1α and (**e**) HIF-2α in HeLa cell lysates from normoxic and hypoxic conditions at the indicated time points were analysed by western blotting with tubulin as loading control. (**f**) FACS quantification of the endocytosed proteome at 2 h of hypoxia in HIF-1α KD (using two different HIF-1α siRNA sequences, as indicated) and control HeLa cells transfected with a scrambled siRNA sequence (Neg Ctrl) shows no significant effect of HIF-1α. Data are presented as % of total cell-surface biotinylation±s.d. (*n*=3). (**g**) HeLa and U87-MG cell lysates from normoxic and hypoxic conditions (2 h) were analysed for caveolin-1 by western blotting with tubulin as loading control. (**h**) Quantification of caveolin-1 to tubulin ratio in hypoxic versus normoxic cells (set to 1). Data represent the average±s.d. from three independent experiments. *P<0.05 (Student's *t*-test). (**i**) Normoxic and hypoxic (2 h) cells were stained for caveolin-1, and filipin as a cell membrane counter-stain. Cell per cell analysis of peripheral, plasma membrane associated caveolin-1 as a fraction of total caveolin-1 was performed using Image J. Data are presented as the fraction of peripheral caveolin-1 per cell in random fields (*n*=51 per condition) in hypoxia relative to normoxia (set to 1) from three independent experiments. *P<0.05 (Student's *t*-test). (**j**) Shown are representative images of the experiment quantified in (**i**). Scale bar, 20 μm.

Caveolin-1 has been reported to regulate both the dynamin-dependent and -independent endocytic pathways^16^,^17^,^18^,^19^,^20^,^21^,^22^,^27^, and we next addressed which of these major pathways are affected. We found that dynamin inhibition in normoxic cells results in a reduction of global protein internalization that is similar to the reduction induced by hypoxia, and that combined treatment with hypoxia and dynamin inhibition did not result in additive reduction of membrane protein internalization as compared with the respective treatments alone (Fig. 4a). Importantly, in both HeLa and MEF cells, the increased protein internalization in hypoxic cells on caveolin-1 deficiency was reversed by dynamin inhibition (Fig. 4b,c). These results provide evidence that the pathway affected by hypoxia is a dynamin-dependent endocytic pathway negatively regulated by caveolin-1.

The above data (Fig. 2d–f) show an effect also of acute hypoxia (2 h) on endocytosis, and the induction of total caveolin-1 even at prolonged hypoxia was significant but limited (Fig. 3d,e), that is, it remained unclear how hypoxia could have such a rapid impact on endocytosis. It has been reported that hypoxic induction of caveolin-1 depends on hypoxia inducible factor (HIF)-1α and HIF-2α and that HIF promotes the formation of caveolae^8^. We found that HIF-1α is substantially induced both at acute (2 h) and prolonged hypoxia (20 h) (Fig. 4d), whereas hypoxic induction of HIF-2α is virtually absent at 2 h and limited at 20 h of hypoxia (Fig. 4e). We thus further explored the potential role of HIF-1α in hypoxic regulation of membrane protein endocytosis. Short interfering RNA (siRNA)-mediated HIF-1α KD using two different siRNA sequences (see Supplementary Fig. 3e for KD validation results) showed no effect on endocytosis in hypoxic cells (Fig. 4f). These data indicated a HIF-1α-independent mechanism and, accordingly, total caveolin-1 levels were not increased in acute hypoxia (Fig. 4g,h). Previous studies on how caveolin-1 can negatively regulate endocytosis independently of caveolae through the formation of stable, oligomerised microdomains or scaffolds at the membrane^17^,^27^ suggested that hypoxia may affect the cellular distribution of caveolin-1. Interestingly, using fluorescence microscopy and quantitative image analysis, we found that the fraction of peripheral, membrane associated caveolin-1 is significantly increased in short term hypoxic (2 h) as compared with normoxic cells (Fig. 4i,j). This changed pattern of caveolin-1 distribution by hypoxia was also consistently found in MEF cells (Supplementary Fig. 3f). To corroborate these results and to exclude the possibility of an immunostaining artefact, we next introduced caveolin-1-mCherry into caveolin-1 KD cells. Real-time confocal imaging clearly showed increased peripheral caveolin-1-mCherry in acute hypoxia (2 h) as compared with normoxic cells (Supplementary Movies 2 and 3; Supplementary Fig. 3g and h). Altogether, these data show that acute hypoxia inhibits endocytosis in a HIF-independent manner, and distributes caveolin-1 to the cell periphery with effects on the dynamics and endocytic capacity of cell-surface proteins.

### Encoding the hypoxic internalizing proteome

**Figure 5:**
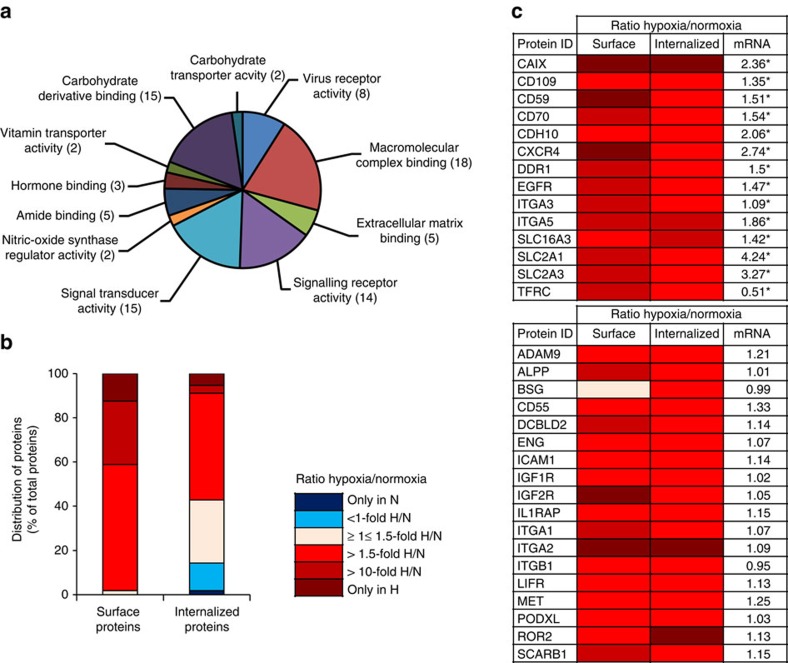
Encoding of hypoxia-induced internalizing surface proteins. (**a**) HeLa cells were pre-treated at normoxia or hypoxia for 20 h. Total surface proteins and internalized proteins, following 2 h of endocytosis, were isolated by streptavidin affinity chromatography and identified by LC–MS/MS analysis. Candidate proteins of interest were selected for label-free MS1 quantification using the Skyline software. MS1 full scan filtering and quantification of peptides identified hypoxia-induced surface proteins (*n*=55) classified into the indicated groups. Data were obtained using the ConsensusPathDB interaction database. (**b**) Quantitative data of candidate proteins at the surface and following internalization. Shown is the distribution of proteins (% of total candidate proteins) according to fold change of protein expression in hypoxic versus normoxic HeLa cells (see also, Supplementary Data 1, 2, 3 and Supplementary Table 1). (**c**) Colour map of relative ratios of 32 candidate proteins at hypoxic versus normoxic conditions, isolated at the cell-surface and following internalization. The upper panel shows proteins with significant, hypoxic regulation of corresponding mRNAs. Gene expression data (right column) are presented as fold increase in hypoxic versus normoxic HeLa cells from three independent experiments. *P<0.05 (Student's *t*-test). See Supplementary Data 3 for full protein names of abbreviations.

We were interested to explore the existence of unique surface antigens that, in contrast to the global trend, may display enhanced internalization at hypoxia. Such proteins should represent interesting candidates for targeting of the tumour microenvironment. To further improve the separation of internalized biotinylated proteins from residual, surface-resident proteins we introduced another blocking step with free streptavidin before isolation of internalized proteins. As shown in Supplementary Fig. 4a–d, using this approach we achieved a complete eradication of remaining surface biotin labelling without affecting labelling of internalized proteins. For proteomic analyses, biotinylated proteins either at the cell-surface or following 2 h of endocytosis from hypoxic and normoxic cells were isolated by streptavidin affinity chromatography. Here, we again took advantage of the cleavable biotin-protein linker to release biotinylated proteins from streptavidin by the reducing agent MesNa. Peptides from eluted proteins were then separated by hydrophobic liquid chromatography and analyzed by tandem mass spectroscopy (LC/MS–MS). Protein identities were selected for further quantitative analyses primarily on the basis of: 1) Their relative absence in negative control cells, 2) Reproducible identification of at least two unique peptides between replicates and 3) Their expected subcellular localization from available literature and open-source protein databases (PubMed and neXtProt) (Supplementary Data 1). Using these criteria, candidate proteins of interest were subjected to MS1 filtering protocol using the publicly available software Skyline (http://proteome.gs.washington.edu/software/skyline)^28^, which has been established as a sensitive approach for high throughput label-free quantification of peptides^29^. Notably, these techniques show variation in peptide signal intensities depending on protein abundance^30^, which is also common for other protein quantification assays^31^. Raw data generated within Proteome Discoverer was imported into Skyline for quantitative analysis on the basis of extracted ion chromatograms from multiple experiments (Supplementary Data 2). From these analyses, we obtained a mapping of relative levels of cell-surface and internalized proteins in hypoxic versus normoxic cells (Supplementary Data 3). When classified according to function by ConsensusPathDB interaction database^32^, most of the candidate proteins were associated with cell signalling and carbohydrate recognition as well as macromolecular binding, in addition to virus receptor activity and extracellular matrix binding (Fig. 5a). As shown in Fig. 5b, the majority of hypoxia regulated proteins were induced 1.5- to 10-fold as compared with normoxia, both at the surface and following internalization, but we also found proteins induced >10-fold or that were exclusively detected in hypoxic cells. Moreover, we found several proteins to be unaltered or even enriched by hypoxia at the surface, but down-regulated in hypoxic as compared with normoxic cells following endocytosis, such as CD44, CXADR, FOLR1, ITFG3, MAGED2, NPR1, PARK7, SRPRB, SLC43A3 and SLC44A1 (Supplementary Data 3). We next focused on proteins of interest that may exhibit enhanced internalization at hypoxia. Out of 55 proteins exhibiting a ≥1.5-fold induction by hypoxia at the surface, 31 proteins also showed a ≥1.5-fold increased internalization in hypoxic as compared with normoxic cells. Gene array analyses revealed that 21 of the hypoxia-induced surface proteins were significantly increased also at the mRNA level (Supplementary Data 3 and Fig. 5c). The validity of our data was supported by the identification of several membrane proteins with enhanced internalization at hypoxia previously found at increased levels in hypoxic or ischaemic cells and tissues, such as CAIX, CD70, CXCR4, EGFR, ENG/CD105, ITAG3, ITAG5, ITGB1, MET, ROR2, SLC2A1/GLUT-1, SLC2A3/GLUT-3 and SLC16A3/MCT-4 (Supplementary Table 1). However, we also found several internalizing proteins that, to our knowledge, are not an established part of the hypoxic response, for example, ALPP, CD59, CD109, CDH10, DDR1, IGFR1, IGF2R, IL1RAP, LIRF, PODXL and SCARB1.

**Figure 6:**
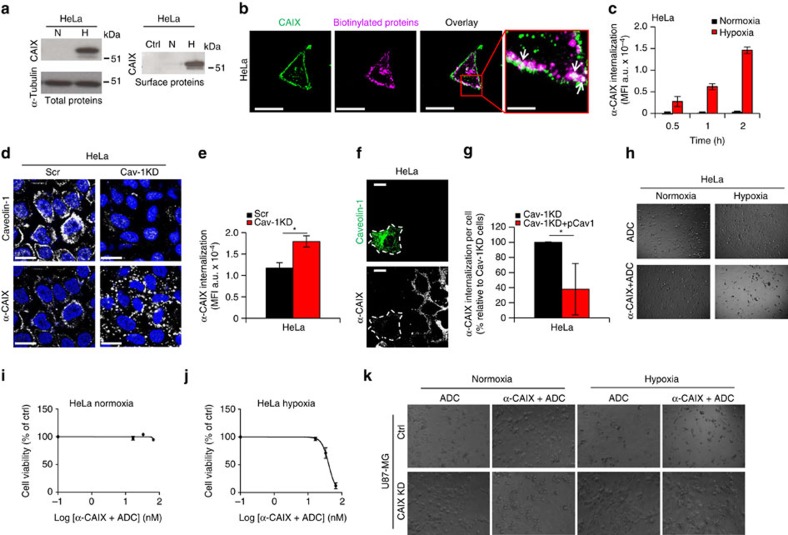
Hypoxia-induced CAIX overrides caveolin-1 negative regulation to allow specific cytotoxin delivery to hypoxic cells. (**a**) Left panel: HeLa cells pre-treated at normoxia or hypoxia for 20 h were analysed for CAIX by immunoblotting with tubulin as loading control. Right panel: HeLa cells were pre-treated as above, followed by cell-surface protein biotinylation and visualization of biotinylated CAIX. Negative control (Ctrl) represents non-biotinylated cells. (**b**) HeLa cells were surface biotinylated, followed by endocytosis for 30 min. Cells were stained for internalized proteins by streptavidin-AF-546 (magenta) and CAIX (green). Right panel shows CAIX co-localization with internalized proteins. Scale bar, 20 μm; right panel, 5 μm. (**c**) HeLa cells were pre-treated as in (**a**) and analysed by FACS for anti-CAIX antibody (α-CAIX) uptake at the indicated time points. (**d**) Caveolin-1 knockdown (Cav-1 KD) and control HeLa cells transduced with scrambled shRNA (Scr) were cultured at hypoxia for 20 h, and α-CAIX internalization and caveolin-1 expression were analysed by confocal microscopy. Scale bar, 20 μm. (**e**) FACS quantification from similar experiment as in (**d**). (**f**) Hypoxic Cav-1 KD cells transiently transfected with caveolin-1-expressing plasmid (pCav-1) were analysed for α-CAIX endocytosis, showing decreased internalization in caveolin-1 overexpressing cells (white dashed line). Scale bar, 20 μm. (**g**) Quantitative results from experiment in (**f**) using Cell Profiler, expressed as % relative to nontransfected Cav-1 KD cells±s.d. from six representative areas (*n*=3). *P<0.05 (Student's *t*-test). (**h**) HeLa cells were pre-incubated in normoxia or hypoxia for 20 h and then treated with α-CAIX pre-complexed with toxin-conjugated IgG (ADC) or ADC alone for another 48 h in normoxia or hypoxia. (**i**,**j**) Similar experiment as in (**h**) showing dose–effect curve of α-CAIX+ADC complex in normoxic (**i**) and hypoxic (**j**) cells. (**k**) CAIX KD and Ctrl U87-MG cells were cultured at normoxia or hypoxia and treated with α-CAIX+ADC complex or ADC alone for 72 h. Data are presented as mean fluorescence intensity (MFI) (a.u.) (**c**,**e**) or as % cell viability of Ctrl (**i** and **j**)±s.d. Images shown in (**b**), (**h**) and (**k**), are representative of at least three independent experiments.

From the list of candidate target proteins (Supplementary Data 3 and Fig. 5c), we were particularly interested in CAIX that by the present approach was exclusively detected in hypoxic cells, both at the surface and following internalization. CAIX is an established hypoxia-responsive protein involved in the adaptive response to intracellular acidosis^33^,^34^,^35^, and we found CAIX messenger RNA (mRNA) to be upregulated by 2.4-fold at 6 h (Fig. 5c) and 7.1-fold at 20 h (Supplementary Fig. 4e) of hypoxia as compared with normoxia. In line with the above results and previous studies, total cell-associated CAIX and surface biotinylated CAIX protein were virtually absent in normoxic while clearly detectable in hypoxic cells, as determined by immunoblotting (Fig. 6a) and FACS (Supplementary Fig. 4f). We found a close co-association between CAIX and internalized biotinylated proteins by confocal imaging (Fig. 6b), and an almost linear increase of anti-CAIX antibody uptake in hypoxic cells over 2 h of incubation, whereas there was no significant uptake in normoxic cells (Fig. 6c). Moreover, we found that CAIX internalization is significantly induced in caveolin-1 KD as compared with control cells, as determined by confocal imaging and quantitative FACS (Fig. 6d,c). Further, this effect was efficiently reversed by reintroducing caveolin-1 into caveolin-1 KD cells (Fig. 6f,g). These results lend further support to the role of caveolin-1 in hypoxic regulation of membrane proteome internalization.

From a tumour targeting perspective, however, it was of interest that the strong hypoxic induction of CAIX internalization can override the negative regulation by caveolin-1. As proof of principle of specific ADC-based targeting of hypoxic cells via CAIX, we next pre-complexed primary anti-CAIX antibody with an antibody conjugated with duocarmycin, that is, a bacteria-derived DNA alkylating agent currently under development for ADC-based treatment of cancer^36^. ADC alone had no effect on the viability of either normoxic or hypoxic HeLa and U87-MG cells (Fig. 6h,k). The anti-CAIX–ADC complex did not affect the viability of normoxic HeLa and U87-MG glioblastoma cells, whereas corresponding cells were efficiently killed by the antibody complex at hypoxic conditions (Fig. 6h–k). To further corroborate antibody target specificity, we next performed experiments with control and CAIX KD cells generated by lentiviral shRNA transduction (Supplementary Fig. 4g). From these experiments, it was clear that anti-CAIX–ADC complex mediated killing of hypoxic cells was dependent on intact CAIX expression (Fig. 6k). We conclude that several surface proteins, in contrast to the global inhibitory effect of hypoxia, show increased internalizing activity in hypoxic cells, thereby constituting potential targets for specific drug delivery to and killing of hypoxic cancer cells. We exemplify this concept by targeting one of the identified candidate proteins, CAIX, with antibody-toxin complexes, showing selective killing of hypoxic cells.

## Discussion

The general principle of targeted cancer therapy is to achieve tumour-specific drug delivery and to reduce unwanted side-effects in healthy tissues. Until now, this concept is best exemplified by the growth factor receptor HER2, that is an established target of the ADC ado-trastuzumab-emtansine in the treatment of breast cancer patients^13^. Improved strategies for the comprehensive analysis of surface protein expression and uptake activity in cancer cells should thus have important clinical implications^37^,^38^. Here, we have implemented a widely applicable method for the comprehensive visualization, quantification and identification of internalizing cell-surface proteins. Using this strategy, we provide new insights into how hypoxia at the global level down-regulates the capacity of cancer cells to endocytose membrane proteins. We provide first evidence that hypoxia down-regulates constitutive membrane protein internalization through mechanisms involving caveolin-1, that is, a key player in clathrin-independent endocytosis. Our data suggest that hypoxia regulates both the total expression and the cellular distribution of caveolin-1 to inhibit endocytosis. Genetic loss of function experiments revealed that hypoxic down-regulation of membrane protein internalization depends on intact caveolin-1 expression. We further demonstrate that caveolin-1 overexpression profoundly restricts the internalizing capacity of hypoxic cells at the global level.

Caveolin-1 is a major structural component of caveolae, which are small (50–100 nm) membrane compartments associated with the plasma membrane^39^ where they play important pathophysiological roles related to signalling transduction and endocytosis with documented implications in tumour development^16^,^17^,^18^,^19^,^20^. Of relevance for the present investigation, caveolin-1 has been shown to attenuate the uptake of a wide variety of ligands, both through the dynamin-dependent and -independent membrane raft pathways^16^,^17^,^18^,^19^,^20^,^21^,^22^,^23^,^24^,^25^,^27^,^40^. We found that dynamin inhibition at normoxia mimics the hypoxic inhibition of protein internalization, and that there is no additive reduction of endocytosis by dynamin inhibition in hypoxic cells. Further, dynamin inhibition attenuated the increased protein internalization in caveolin-1-deficient cells. These results show that caveolin-1 negatively regulates dynamin-dependent endocytosis in hypoxic cells. Recent studies suggest that caveolin-1 may have direct modulatory effects on plasma membrane lipid and protein organization that is not directly related to caveolae formation^17^,^27^,^41^,^42^. Specifically, the caveolin-1 scaffolding domain was linked to perturbed diffusion within membrane lipid microdomains, resulting in decreased membrane protein mobility^43^. The previous and present investigations point at the interesting possibility that cellular uptake of microbes, exosomes and other macromolecular ligands is governed by local oxygen availability and hypoxia-mediated regulation of caveolin-1 expression and cellular distribution with general relevance in pathophysiology.

Cellular adaptation to hypoxia is largely executed by HIFs, which are heterodimeric transcription factors composed of a constitutively expressed β-subunit and one of three hypoxia inducible HIF-α-subunits^44^. Interestingly, Wang *et al*.^8^, reported that ligand-independent activation of the receptor tyrosine kinase (RTK) EGFR, that is, one of the candidate proteins showing enhanced internalization at hypoxia (Fig. 5), was enhanced due to HIF-mediated caveolin-1 and caveolae induction. Further, in the context of ligand-dependent EGFR internalization, hypoxia was shown to maintain EGFR signalling to promote cell survival by decelerating Rab5-mediated fusion of early endosomes^7^. However, we found that hypoxia can inhibit global protein internalization independently of HIFs. Additional RTKs associated with tumour development may be hypoxia-regulated^9^, such as the hepatocyte growth factor receptor MET^10^. Notably, we identified several RTKs, including MET, as candidate proteins exhibiting increased constitutive internalization under hypoxic conditions (DDR1, IGFR1, IGF2R and ROR2; see Fig. 5 and Supplementary Table 1). Thus, while causing a general down-regulation of membrane protein expression and internalization, hypoxia may selectively induce constitutive RTK internalization and signalling in a situation of poor ligand availability, thereby promoting tumour progression and development. We also identified several members of the integrin family (ITGA1, -2, -3 and -5; ITGB1 and -5) among our candidate proteins. Endosomal sorting and recycling of integrins have been shown to regulate cancer cell adhesive and migratory behaviour^45^ with important implications for tumour development and metastasis^3^. How caveolin-1 may act in concert with additional regulators of membrane protein mobility to regulate the spatial distribution of, for example, integrins and RTKs in the hypoxic tumour microenvironment should be an interesting area of future investigations.

The first established example of direct targeting of the hypoxia-induced pathways in clinical oncology is represented by anti-vascular endothelial growth factor antibody therapy with bevacizumab; however, the success of anti-angiogenic agents may be limited by the selection of malignant cells that adapt to an even more hypoxic microenvironment. Our results support the concept that the adaptive response of cancer cells to hypoxic stress provides opportunities for tumour-specific drug delivery via hypoxia-induced internalization of specific membrane proteins. Notably, we show that CAIX induction can override the negative regulation by caveolin-1 on protein internalization in hypoxic cells. As proof of principle of this concept, we show that an ADC complex targeted at CAIX is efficiently and specifically internalized at hypoxic conditions, resulting in highly selective toxin delivery to and killing of hypoxic cells. In summary, our data show that hypoxia and caveolin-1 at a functional level regulates the internalizing activity of the membrane proteome. These findings have potential implications for the spatial regulation of the receptor signalling pathways in tumour biology as well as for the development of treatment strategies specifically targeted at the tumour microenvironment.

## Methods

### Cell-lines and clinical samples

Patient derived cancer cell-lines HeLa (cervical carcinoma), U87-MG (glioblastoma multiforme), MDA-MB-231 (breast adenocarcinoma), A549 (lung adenocarcinoma), and WT and caveolin-1 KO MEF were newly purchased from ATCC at the initiation of the described studies. Tumour specimens were obtained from patients with primary glioblastoma (World Health Organization grade IV) at the Department of Neurosurgery, Skåne University Hospital, Lund. Biopsies were collected with informed consent according to Protocol H15 642/2008 approved by the Lund University Regional Ethics Board, Lund, Sweden.

### Cell culture

Cells were routinely cultured in DMEM, 4.5 g l^−1^ glucose (Thermo Scientific), supplemented with 10% foetal bovine serum (FBS, Sigma-Aldrich), 1% penicillin–streptomycin (Sigma-Aldrich), 1% L-glutamine (Sigma-Aldrich) at 37 °C in a humidified 5% CO_2_, 21% O_2_ atmosphere. Hypoxic conditions were obtained in a Sci-tive-NN cell work station (Ruskinn Technology Ltd) at 37 °C, in a humidified 5% CO_2_, 1% O_2_ atmosphere.

### Lentiviral shRNA and siRNA KD and transfectants

HeLa and U87-MG caveolin-1 and CAIX KD cells were generated using the Mission TRC lentiviral transduction particles (SHCLNV, Sigma-Aldrich) according to the manufacturer's instructions. Briefly, cells were infected with either caveolin-1 shRNA (NM_001753; TRC0000011218), CAIX shRNA (NM_001216; TRCN0000150123) or a negative control, scrambled sequence (SHCO16V, pLKO.1-puro) at a multiplicity of infection (MOI) of 0.5 for 48 h. Stable HeLa and U87-MG clones were obtained by further culturing in medium containing 3.5 and 32 μg ml^−1^ puromycine (Sigma-Aldrich), respectively. For transient siRNA KD, cells were transfected with 50 nM Silencer Validated HIF-1α siRNA (Ambion, #42840) or nonspecific siRNA (Ambion negative control siRNA #1), or 200 nM Silencer Select Validated (Ambion, #s6539) using Lipofectamine 3000 reagent (Invitrogen). Following an incubation period of 24 h in fresh growth medium, cells were transferred to 24-well plates for further analyses. For transient transfections, cells were electroporated (ECM 399, BTX Harvard Apparatus) with 30 μg pcDNA3.1/ myc-his C plasmid encoding Myc-tagged human caveolin-1 kindly provided by Dr Jeffrey E. Pessin (Stony Brook University, New York)^46^, or transfected using Lipofectamine 2000 (Invitrogen) with CAV1-mCherry encoding plasmid (a gift from Ari Helenius; Addgene plasmid # 27705)^47^, followed by seeding in Lab-Tek II Chambered Coverglass (Nunc) at approximately 125,000 cells cm^–2^ in DMEM containing 20% FBS, 4.5 g l^−1^ glucose, 1% penicillin–streptomycin, 1% L-glutamine, for 24 h before further analyses.

### Cell-surface proteome biotinylation

Normoxic or hypoxic subconfluent cells were pre-incubated on ice for 10 min and maintained on ice during the following steps to prevent internalization. Cells were washed with ice-cold PBS containing MgCl_2_ and CaCl_2_ (Mg/Ca–PBS; Thermo Scientific) adjusted to pH 8, followed by incubation with 1 mg ml^−1^ of a membrane impermeable and cleavable biotin moiety (EZ-Link Sulfo-*N*-hydroxysuccinimide-SS-Biotin, Thermo scientific) in Mg/Ca–PBS for 30 min, and washed with ice-cold Mg/Ca-PBS. Free biotin was quenched with 0.1 M glycine in Mg/Ca–PBS for 10 min, and cells were subsequently washed with ice-cold Mg/Ca–PBS before further analyses.

### Endocytosis of biotinylated cell-surface proteins

Following cell-surface biotinylation, endocytosis was initiated by the addition of pre-warmed SF medium for the indicated time periods at 37 °C in a 21% or 1% O_2_ atmosphere. Endocytosis was stopped by incubation on ice for 10 min. For removal of cell-surface biotin, cells were incubated with 300 mM MesNa (sodium-2-mercaptoethanesulfonate; Thermo Scientific) in 50 mM Tris pH 8.6 containing 100 mM NaCl, 1 mM EDTA and 0.2% BSA in three consecutive steps at 4 °C in the dark. Cells were rinsed with ice-cold Mg/Ca–PBS and then incubated with iodoacetamide (5 mg ml^−1^, Sigma-Aldrich) in Mg/Ca–PBS for 10 min in dark, and subsequently washed with Mg/Ca–PBS. For LC–MS/MS experiments, an additional cell-surface biotin blocking step was included with free streptavidin (25 μg ml^−1^ or as indicated; Sigma-Aldrich) diluted in Mg/Ca–PBS containing 1% BSA for 30 min at 4 °C, followed by extensive washing to remove unbound streptavidin.

### FACS quantification

Surface and internalized protein biotinylation was quantified on a per cell basis after cell detachment, fixation with 2% paraformaldehyde and permeabilised with 0.5% saponin in PBS. Nonspecific binding was blocked with 3% BSA in PBS before labelling with 5 μg ml^−1^ streptavidin-Alexa Fluor (AF)-488 (S32354, Life Technologies) in Mg/Ca–PBS containing 3% BSA for 30 min at 4 °C. Data were acquired on an Accuri C6 flow cytometer and analysed using Accuri C6 software (BD Biosciences). Results were expressed after subtraction of the values of negative control cells (no biotinylation+streptavidin-AF-488 labelling). The internalized protein–biotin pool was expressed as percentage of the total cell-surface protein–biotin signal after subtraction of residual biotin after MesNa treatment. In some cases, cells were pre-treated prior to biotinylation, that is, with Dynasore (100 μM, 30 min; Sigma-Aldrich), methyl-β-cyclodextrin (5 mM, 1 h; Sigma-Aldrich), human low-density lipoproteins (100 μg ml^−1^, 20 h; Intracel), U0126 (10 μM, 1 h; Selleck Chemicals) or Wortmannin (240 nM, 1 h; Sigma-Aldrich) in SF medium. Constitutive endocytosis was induced for 30 min in the continuous presence of inhibitors.

Surface labelling of CAIX was performed by incubating detached cells (normoxic or hypoxic) with M75 anti-CAIX antibody^48^ (M75, Bioscience Slovakia) for 1 h at 4 °C, and then for 30 min with AF-488-conjugated secondary antibody in 1% BSA in PBS. For detection of CAIX internalization, M75 antibody (1/200) was pre-complexed with AF-488 secondary antibody (1/500) (A1101, Life Technologies) in SF medium for 30 min, and then added to adherent cells during the indicated time periods at 37 °C. Cells were detached, washed in 1% BSA in PBS and resuspended in PBS containing 2% formaldehyde and 0.02% sodium azide, and analysed by flow cytometry.

### Ligand uptake experiments

Cells were incubated with 10 μg ml^−1^ of Transferrin-Alexa-488, 5 μg ml^–1^ of Cholera Toxin subunit B-Alexa-488 (Life Technologies), or 500 μg ml^−1^ of Dextran-FITC (Sigma-Aldrich) during 30 min at 37 °C in SF medium. Cells were detached, washed in PBS and resuspended in PBS containing 2% formaldehyde (Sigma) and 0.02% sodium azide (Merck). Cell-associated fluorescence was acquired on an Accuri C6 Flow cytometer and analysed using Accuri C6 software (BD Biosciences).

### Endocytosis treatments

Cells were treated with Dynasore (100 μM, 30 min; Sigma-Aldrich), methyl β-cyclodextrin (5 mM, 1 h; Sigma-Aldrich), human low-density lipoproteins (100 μg ml^−1^ 20 h; Intracel), U0126 (10 μM, 1 h; Selleck Chemicals) or Wortmannin (240 nM, 1 h; Sigma-Aldrich) in SF medium. Dynasore, Wortmannin and U0126 were maintained during 30 min of endocytosis.

### Immunofluorescence imaging

Surface and internalized biotinylated proteins were visualized after fixation with 2% PFA for 10 min, and permeabilised for 30 min with 0.5% saponin diluted in PBS. Nonspecific sites were blocked with PBS containing 3% BSA before labelling with 20 μg ml^−1^ Streptavidin-AF-488 or -633 (S32352, S21374, Life Technologies) in PBS, 3% BSA for 30 min at 4 °C. For immunofluorescence visualization, cells were incubated with anti-Caveolin-1 (ab2910; 1/1500; Abcam), anti-CAIX (M75; 1/200; Bioscience Slovakia) primary antibody for 1 h at room temperature or anti-early endosome antigen (EEA1) (ab2900 (1/100; Abcam) antibody overnight at 4 °C. After washing, cells were incubated with AF-488 or -546-conjugated secondary antibody (1/500) (A11001, A11060, Life Technologies) for 1 h at 4 °C and nuclei were counterstained with Hoechst 33342 (1/20,000) (1399, Life technologies) for 30 min. The acquisition of images was performed using Zeiss LSM 710 confocal scanning microscope equipped with excitation laser wavelengths of 405, 488 and 633 nm, and a C-Apochromat 63X/1.20 W korr M27 water or Plan-Apochromat 63 × /1.40 DIC M27 oil immersion objective. Images were acquired using Zen 2011 software (Carl Zeiss). Cell per cell quantification of internalized proteins in caveolin-1 KD and overexpressing cells was performed using Cell profiler 2.0 software (The Broad Institute, Boston) by identification of cell cytoplasm and measuring per-cell dot intensity on random fields (*n*=6) from three independent experiments. Cell per cell analysis of peripheral, plasma membrane associated caveolin-1 as a fraction of total caveolin-1 was performed on cells stained for caveolin-1, and filipin-III (Sigma-Aldrich) as cell membrane counter-stain, using Image J on random fields (*n*=51) from three independent experiments. Human glioblastoma tumour cryosections were fixed with ice-cold 99% ethanol and blocked in PBS with Tween 20 and 5% FBS, followed by incubation overnight at 4 °C with primary antibodies to GLUT-1, ab40084 (1/100; Abcam) and caveolin-1, ab2910 (1/100) in blocking buffer, and then with AF-488 or -546-conjugated secondary antibodies (1/300) for 1 h at room temperature. Sections were mounted with PermaFluor (Beckman Coulter), and analysed using Axio Observer.Z1 HB 100 fluorescence microscope equipped with a 20 × water objective (Carl Zeiss).

### Real-time confocal microscopy imaging

Surface protein biotinylated cells, as described above, were incubated on ice with AF-488-streptavidin for 30 min in Mg/Ca–PBS. After extensive washing, endocytosis was induced by adding pre-warmed SF medium, and cells were transferred to a humidified 5% CO_2_, 21% O_2_ atmosphere incubator integrated with Zeiss LSM 710 confocal scanning equipment. Internalized, biotinylated proteins and caveolin-1-mCherry transfected cells were visualized in phenol red-free medium without serum using laser excitation at 488 and 546 nm, respectively, and EC Plan-Neofluar 40 × /1.30M27 or C-Apochromat 63X/1.20WM27objectives. Images were collected during a time series of 60 min with 5 s cycle time (biotinylated proteins) or 3 min with 5 s cycle time (caveolin-1-Cherry).

### Isolation of biotinylated proteins

Biotinylated cells from hypoxic and normoxic conditions with and without constitutive endocytosis as described above were lysed for 20 min at 4 °C in RIPA buffer (8 μl cm^–2^) containing 10 mM Tris pH 7.4, 150 mM NaCl, 1 mM EDTA, 0.1% SDS, 1% Triton X-100 and 1% sodiumdeoxycholate, protease inhibitors (Complete; Roche Diagnostics) and phosphatase inhibitors (Roche Diagnostics). Lysates were clarified by centrifugation at 18,000*g* for 10 min at 4 °C. The soluble fraction was collected and total protein was quantified using BCA Protein assay kit (Pierce). Lysates from control and biotinylated cells were diluted 1:4 with Mg/Ca–PBS supplemented with protease inhibitors and applied to a HiTrap streptavidin HP-1 ml column (GE Healthcare) pre-equilibrated with 10 column volumes of PBS 0.1% Triton X-100 using a peristaltic pump set at a flow rate of 250 μl min^−1^. The column was then transferred to an HPLC UPC 900 system (Amersham Biosciences) with an online UV detector set at 280 nm, and washed with 12 ml of PBS 0.1% Triton X-100, followed by 10 ml of RIPA/PBS 0.1% Triton X-100 1 M NaCl 1:1 (v/v), and finally 10 ml of PBS 0.1% Triton X-100 to remove non-biotinylated proteins. Biotinylated proteins were then eluted from the column by reduction of the protein-SS-Biotin linker with 10 ml 150 mM MesNa in PBS 0.1% Triton X-100 applied at a reduced flow rate (125 μl min^−1^). One volume of 20% TCA was added to the collected eluate to precipitate proteins by incubation for 30 min on ice and centrifugation for 10 min at 18,000*g*. The pellets were washed with 2% sodium acetate, air dried and resuspended in RIPA for western blot analysis or in 3 M urea buffer for mass spectrometry analysis.

### Mass spectrometry analyses

Protein pellets, isolated as described above, were dissolved in 50 mM ammonium bicarbonate (AMBIC) buffer containing 3 M urea. Protein samples were then reduced with 10 mM dithiothreitol and alkylated using 40 mM iodoacetamide in the dark, followed by buffer exchange with 50 mM ammonium bicarbonate, pH 7.6. Protein samples were digested with sequencing grade trypsin (Promega, Madison) overnight at 37 °C. The digestion was stopped by adding 1% formic acid, samples were dried using a Speed Vac, resuspended in 1% formic acid, and centrifuged for 5 min at 10,000*g*. The supernatants were stored at −80 °C until further analysis. The peptides were analysed by a top 10 method using a Q-Exactive. Full MS scans were acquired in the Orbitrap mass analyzer over *m*/*z* 350–1,800 range with resolution 70,000 (*m*/*z* 200). The target value was 3 × 10^6^ ions. The 10 most intense peaks with charge state ≥2 were fragmented in the higher-energy collision dissociation (HCD) collision cell with normalized collision energy of 30%, and a tandem mass spectrum was acquired in the Orbitrap mass analyzer with resolution 17,500 at *m*/*z* 200. The target value was 1 × 10^5^ ions. The ion selection threshold was 3.30 × 10^5^ counts, and the maximum allowed ion accumulation times were 100 ms for full MS scans and 150 ms for tandem mass spectrum. The total amount of protein digest injected into the MS/MS platform was 1 μg each in three independent experiments. The samples were first loaded onto a trap column (300 μm × 5 mm, C18, 5 μm and 100 Å, Thermo Scientific), and were then separated using a C18 column (50 cm × 75 μm, C18, 2 μm and 100 Å, PN 164540; Thermo Scientific) with a flow rate of 250 μl min^−1^. Solvent A (0.1% formic acid) and solvent B (0.1% formic acid and 100% acetonitrile) were mixed for a nonlinear gradient of 5–20% B over 90 min, followed by 20–40% B over 90 min, and 40–90% B over 95 min. For all the experiments, dynamic exclusion was set to 90 s. Raw data was analysed with Proteome Discoverer v 1.3 (Thermo Scientific) using both Sequest and Mascot search engines. Precursor and fragment mass tolerances were set to 1 Da, where the maximum of missed cleavage sites was two and 1% false discovery rate was used. At least two unique peptides were necessary for protein identification.

### Label-free quantification by MS1 filtering analysis

MS1 chromatogram-based quantification was performed in Skyline version 2.5, http://proteome.gs.washington.edu/software/skyline^28^,^29^. A spectral library was established by importing the MSF files generated within Proteome Discoverer into Skyline. Then, raw data files were directly imported into Skyline, and MS1 precursor ions extracted for all peptides presented in the MS/MS spectral libraries. Quantitative analysis is on the basis of extracted ion chromatograms (XICs) and resulting precursor ion peak areas for each peptides M, M+1 and M+2, that is, the first, second and third isotopic envelope. Peaks with identification annotations were subjected for relative quantification within the sample digests. Integration of the peaks was adjusted when signals were relatively weak and the software could not reliably determine the peaks. Following data extraction, peptide areas of biotinylated samples treated with MesNa (negative control) were subtracted from peak areas of biotinylated samples. The peptide areas were then averaged across triplicates, and a ratio of mean hypoxia intensity divided by mean normoxia intensity was generated for each peptide quantified.

### Immunoblotting

Protein samples were mixed with NuPAGE LDS Sample Buffer 4 × (Life Technologies) and heated at 80 °C for 10 min. Equal amount of proteins were loaded and separated in a NuPage 4–12% Bis Tris gel (Life Technologies) at non-reducing (in the case of Sulfo-*N*-hydroxysuccinimide-SS-Biotin-conjugated proteins) or reducing conditions, then transferred onto a polyvinylidene fluoride (PVDF) membrane, followed by blocking in TBS 0.05% Tween 20 containing 3% BSA, and incubation with Streptavidin-Peroxidase polymer (1/10,000) (S2438, Sigma-Aldrich) in TBS 0.05% Tween 20, 3% BSA. Loading controls were obtained by labelling PVDF membranes with 0.1% Coomassie R350 (GE Healthcare) in 60% methanol in water during 1 min, followed by destaining in 10% acetic acid–50% ethanol in water. Caveolin-1, CAIX, HIF-1α, HIF-2α, α-tubulin and actin were probed after membrane blocking in TBS 0.05% Tween 20 containing 5% milk by incubation with the following primary antibodies overnight at 4 °C: Anti-caveolin-1 ab2910 (1/4,000; Abcam), anti-CAIX M75 (1/200; BioScience Slovakia), anti-HIF-1α #628480 (1/1,00; Genetex), anti- HIF-2α ab199 (1/500; Abcam), anti-α-tubulin ab7291 (1/10,000; Abcam), anti-β-actin ab8227 (1/5000; Abcam), and then incubated with horseradish peroxidase conjugated anti-rabbit (1/10,000) (7074, Cell Signalling Technology) or anti-mouse IgG (1/10,000) (19044, Sigma-Aldrich) secondary antibodies. Protein bands were visualized by enhanced chemiluminescence (ECL) western blotting substrate (Pierce), and their intensities were measured by densitometry using ImageJ software. For uncropped scans of immunoblots, see Supplementary Fig. 5.

### Gene expression microarray analyses

Cells were incubated in SF medium either in 21% O_2_ or 1% O_2_ for 6 or 20 h. Total RNA was isolated with the RNeasy Mini Kit (Qiagen) and quantified with Nanodrop spectrophotometer (Saveen Werner). RNA integrity was verified on Agilent 2100 Bioanalyzer, and microarray experiments were performed at the Swegene Center for Integrative Biology, Lund University, using the Illumina Human HT-12 v4 Expression BeadChip. Data filtration and normalization were performed using BASE2 (ref. 49). Transcripts showing a detection *P*-value <0.01 were further analysed using the R statistical programming environment. Change in gene expression was calculated as a ratio of the mean of hypoxic versus the mean of normoxic values from three separate experiments. The data reported in this paper have been deposited in the Gene Expression Omnibus database, www.ncbi.nlm.nih.gov/geo (accession nos. GSE45301 and GSE79069).

### Antibody-drug conjugate cytotoxicity assay

HeLa (3,000 cells per well) and control U87-MG or U87-MG CAIX KD cells (5,000 cells per well) were seeded in 96-well plates and incubated overnight at 37 °C in 95% humidified atmosphere containing 5% CO_2_. The following day, media were changed to SF medium before incubating them for 20 h at 21% or 1% O_2_, at 37 °C. Anti-mouse CAIX primary antibody (1/100) was added to the appropriate wells followed by the addition of the indicated concentrations of anti-mouse IgG Fc-duocarmycin (αMFc-CL-DMSA; Moradec) to HeLa cells or anti-mouse IgG Fc-monomethyl auristatin F (αMFc-CL-MMAF; Moradec) to U87-MG cells. Cells were then incubated for an additional 48 h (HeLa cells) or 72 h (U87-MG and U87-MG CAIX KD cells), and cell viability was assayed using the MTS reagent by absorbance measurement at 490 nm in a VERSAmax microplate reader with SoftMax Pro Software. The cytotoxicity of antibody-drug conjugates was analysed using GraphPad Prism software v6, and representative cell images were captured on an Axiovert 40C microscope (20 × objective; Carl Zeiss).

### Statistical analyses

Student's *t*-test was employed for the comparison of groups containing three or more replicates. Statistical significance was set at *P*<0.05. Error bars represent s.d.

## Additional information

**Accession codes:** Microarray data have been deposited in the Gene Expression Omnibus (GEO) database, under accession codes GSE45301 and GSE79069.

**How to cite this article:** Bourseau-Guilmain, E. *et al*. Hypoxia regulates global membrane protein endocytosis through caveolin-1 in cancer cells. *Nat. Commun.* 7:11371 doi: 10.1038/ncomms11371 (2016).

## Supplementary Material

Supplementary Information Supplementary Figures 1-5, Supplementary Table 1 and Supplementary References

Supplementary Data 1 Protein Identification list

Supplementary Data 2 Quantification of proteins from surface and internalized biotinylated proteins

Supplementary Data 3 Protein identification table

Supplementary Movie 1 Real-time visualisation of cell-surface proteome endocytosis. HeLa cells were surface biotinylated and recorded during the initial 60 min of membrane protein internalisation by confocal microscopy. Shown is a time series with image capture every 5 s using laser excitation at 488 nm and an EC Plan-Neofluar 40x/1.30M27 Oil immersion objective.

Supplementary Movie 2 Real-time visualisation of caveolin-1-mCherry in normoxic cells. HeLa caveolin-1 KD cells were transfected with caveolin-1-mCherry expressing plasmid and recorded by confocal microscopy. Shown is a time series of 3 min with 5 s cycle time using laser excitation at 546 nm and a C-Apochromat 63X/1.20WM27 objective.

Supplementary Movie 3 Real-time visualisation of caveolin-1-mCherry in hypoxic cells. HeLa caveolin-1 KD cells were transfected with caveolin-1-mCherry expressing plasmid and recorded by confocal microscopy after incubation for 2 h in hypoxia. Shown is a time series of 3 min with 5 s cycle time using laser excitation at 546 nm and a C-Apochromat 63X/1.20WM27 objective.
