# Supplementary material for: Hypoxia regulates global membrane protein endocytosis through caveolin-1 in cancer cells
Source: Nat Commun. 2016 Apr 20;7:11371. doi: 10.1038/ncomms11371 (PMC4842985; doi:10.1038/ncomms11371)
Supplement: Supplementary Information — Supplementary Figures 1-5, Supplementary Table 1 and Supplementary References [file ncomms11371-s1.pdf]

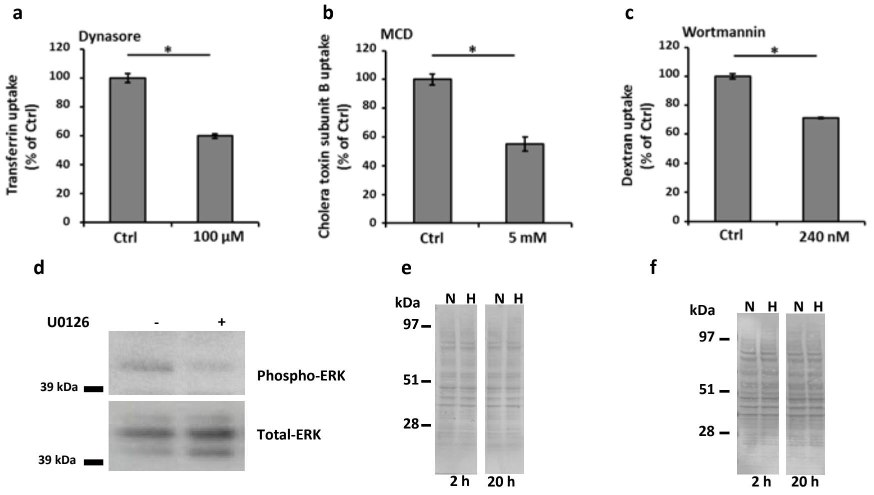

Supplementary Figure 1. Effects of endocytosis inhibitors on their respective targets, and protein loading controls. HeLa cells were pre-treated with the indicated concentrations of dynasore for 30 min (**a**), MCD for 1 h (**b**), or wortmannin for 1 h (**c**), and the effect on clathrin-mediated transferrin, membrane raft-dependent cholera toxin subunit-B and macropinocytic dextran uptake, respectively, was analysed by FACS. Data are presented as % of untreated control cells (Ctrl) ± S.D. from three independent experiments. * $p < 0.05$. **d**) HeLa cells were either untreated or pre-treated with the MEK inhibitor UO126 (10 μM) for 1 h, and analysed by western blotting for phosphorylated ERK1/2 (phospho-ERK1/2) or total ERK1/2 as loading control. **e)** and **f)** Protein loading controls of experiments shown in **Fig. 2c** and **f**, respectively, show equal total protein loading from normoxic (N) and hypoxic (H) HeLa cells.

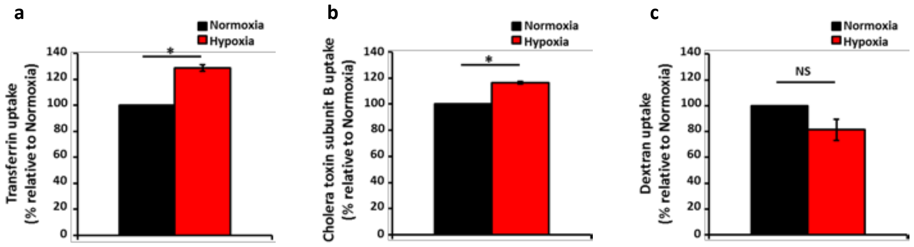

Supplementary Figure 2. Effects of hypoxia on major endocytic pathways. HeLa cells were pre-treated at normoxia or hypoxia (1% O_2) for 20 h, and then analysed for the uptake of (**a**) transferrin (10 μg mL^{-1}), (**b**) cholera toxin subunit-B (5 μg mL^{-1}) or (**c**) dextran (500 μg mL^{-1}) for 30 min by FACS. Data are presented as % of normoxic cells ± S.D. from three independent experiments. * $p < 0.05$.

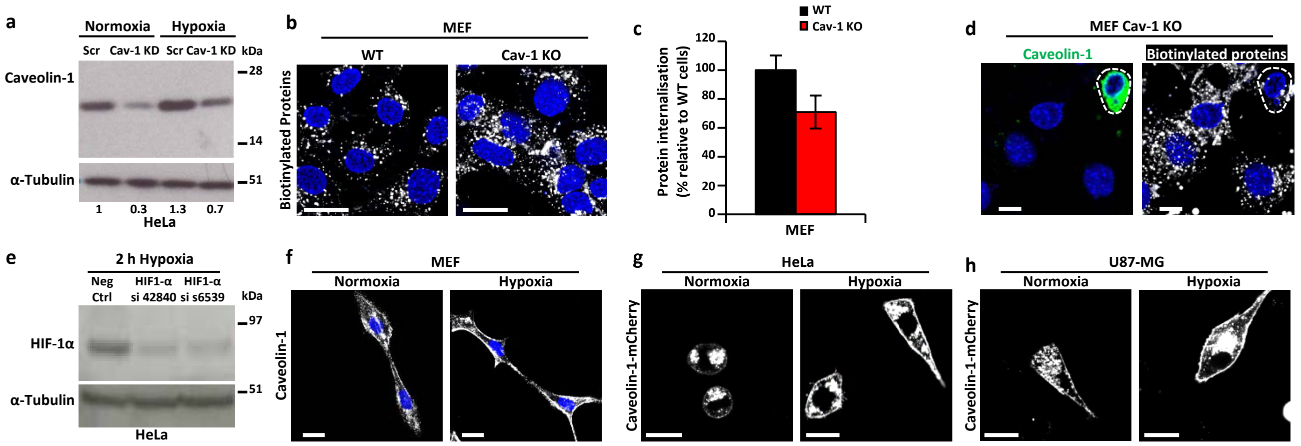

Supplementary Figure 3. Hypoxic regulation of endocytosis and caveolin-1. a) Caveolin-1 knockdown HeLa cells (Cav-1 KD) and control cells transduced with a scrambled shRNA sequence (Scr) from normoxic and hypoxic conditions were analysed by immunoblotting for caveolin-1 with tubulin as loading control. The caveolin-1 to tubulin ratios (normoxic Scr set to 1) from a representative experiment are indicated. **b)** Hypoxic wild-type (WT) and caveolin knockout (Cav-1 KO) MEF cells were surface biotinylated, followed by endocytosis for 30 min and subsequent cell-surface biotinylation depletion. Cells were stained for internalised proteins, showing increased internalisation in caveolin-1 KO as compared with WT MEF cells. Scale bar, 20 μm. **c)** FACS quantification of the endocytosed proteome at 30 min in normoxic WT and Cav-1 KO MEF cells. Data are presented as % relative to WT cells ± SD from two independent experiments (n = 3). **d)** Hypoxic Cav-1 KO MEF cells transiently transfected with caveolin-1 expressing plasmid were surface biotinylated followed by endocytosis for 30 min and subsequent depletion of cell-surface biotinylation. Cells were stained for internalised proteins and caveolin-1, showing decreased internalisation in caveolin-1 overexpressing cells (indicated by white dashed line). Scale bar, 10 μm. **b)** and **d)**, Shown are representative images from three independent experiments. **e)** HIF-1α knockdown

in hypoxic HeLa cells using siRNA transfection with two different sequences (siHIF-1α 42840 and s6539). Control HeLa cells were transfected with a scrambled siRNA sequence (Neg Ctrl). Shown is a representative immunoblotting for HIF-1α and tubulin as loading control. **f)** Normoxic and hypoxic MEF WT cells were stained for caveolin-1, showing increased perpiheral signal in hypoxic as compared with normoxic cells. **g)** HeLa and **h)** U87-MG caveolin-1 KD cells were transfected with caveolin-1-mCherry expressing plasmid, followed by treatment in normoxia or hypoxia for 2 h. Caveolin-1-mCherry cellular distribution was then visualized by confocal microscopy using laser excitation at 546 nm and a C-Apochromat 63X/1.20WM27 objective. Shown are representative images from at least three independent experiments (see also, *Supplementary Movies 2 and 3*). Scale bar, 20 μm.

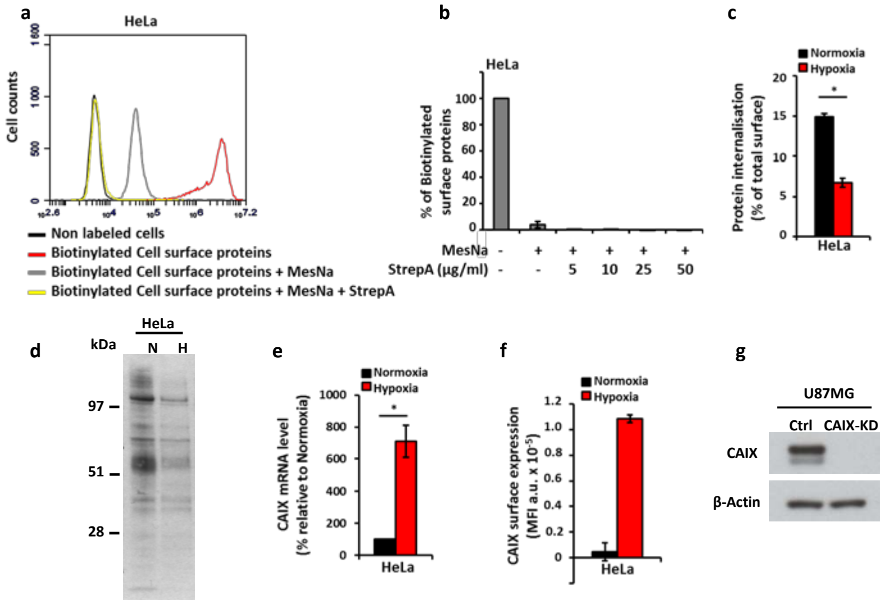

Supplementary Figure 4. Optimised procedure for proteomic analyses of internalised surface proteins; hypoxic induction of CAIX. a) FACS analysis of biotinylated cell-surface proteome in HeLa cells. Shown is a representative experiment of non biotinylated cells, total cell-surface biotinylation, cell-surface signal following reductive cleavage of biotin-protein linker with MesNa, and the complete absence of residual cell-surface signal following reductive cleavage of biotin-protein linker with MesNa and blocking with free streptavidin. **b)** Quantitative analysis from a similar experiment as in (**a**) shows efficient blocking of cell-surface biotinylation by combined MesNa treatment and free streptavidin. **c)** HeLa cells were pre-treated at normoxia or hypoxia (1% O_2) for 20 h, followed by cell-surface biotinylation and endocytosis for 2 h. Residual surface proteins were removed by combined MesNa treatment and blocking with free streptavidin, and internalised proteins were quantified by FACS. Data are presented as % of total cell-surface biotinylation ± S.D. from three independent experiments. * $p < 0.05$. **d)** Immunoblotting for endocytosed proteins from a similar experiment as described in (**c**) shows reduced protein levels in hypoxic as compared with normoxic cells. **e)** CAIX gene expression in hypoxic (20 h of hypoxia) *vs.* normoxic cells presented as % relative to normoxia ± SD from three independent experiments. * $p < 0.05$. **f)** HeLa cells were pre-treated at normoxia or hypoxia (1% O_2) for 20 h and analysed for cell-

surface CAIX expression by FACS. **g)** Stable CAIX knockdown U87-MG cells (CAIX KD) were generated by lentiviral shRNA transduction. CAIX KD and scrambled control (Ctrl) cell lysates from hypoxic conditions were analysed for CAIX by western blotting with β-actin as loading control.

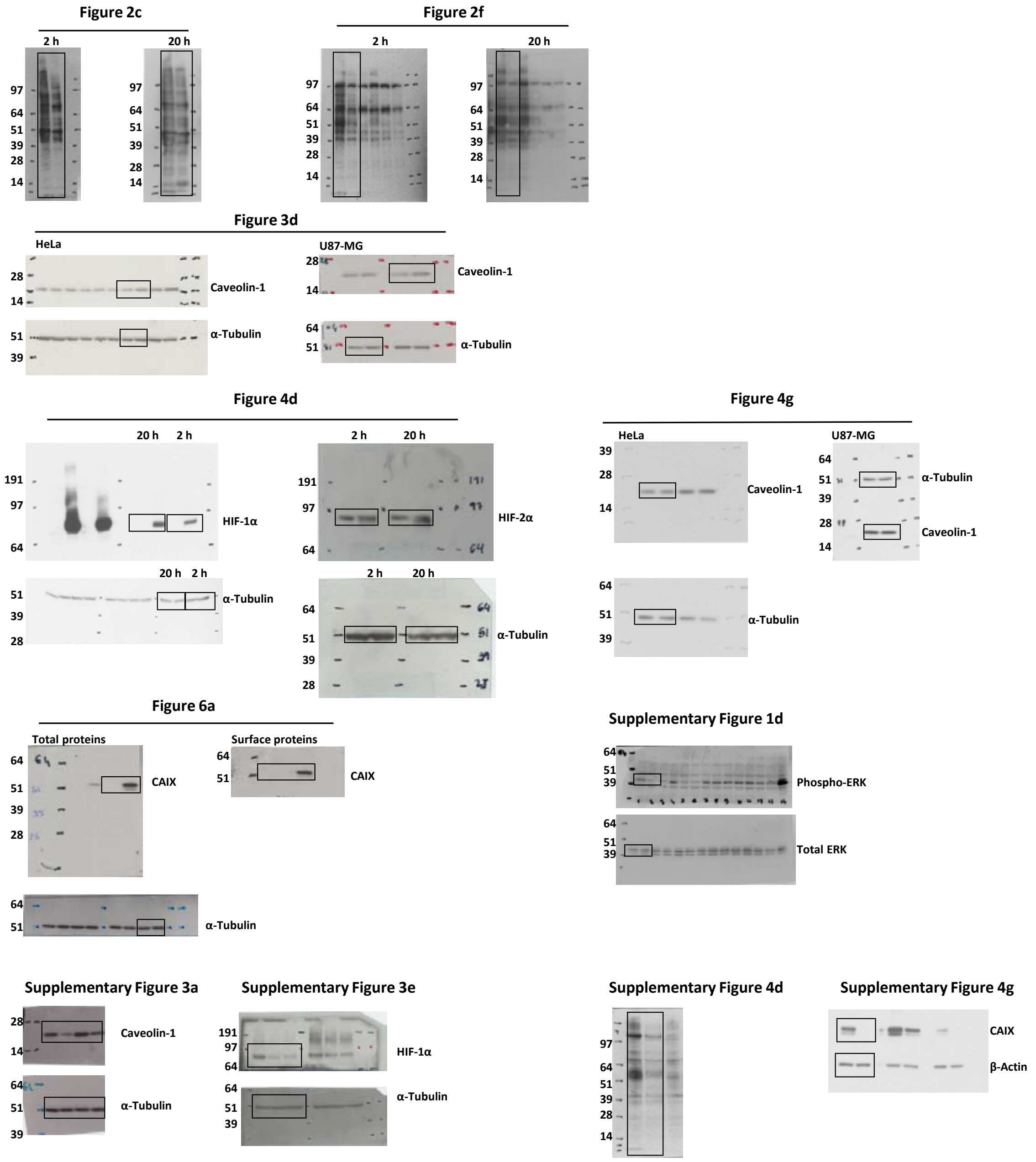

Supplementary Figure 5. Full blots corresponding to portions of blots presented in the respective figures, as indicated.

Supplementary Table 1. Summary of selected membrane proteins showing hypoxia-induced internalisation. See also, Fig. 4.

Protein ID	Potential hypoxia regulation and role in tumour biology	Refs.
**CAIX**	HIF induced; commonly associated with poor patient outcome; part of pH-regulatory machinery; potential target of treatment and PET tumor imaging.	1, 2
**CD59**	Contributes to tumor cell evasion of immune cell attack; potential treatment target.	3, 4
**CD70**	HIF induced; potential treatment target.	5, 6
**CXCR4**	HIF induced; potential treatment target; receptor of non-viral gene delivery.	7-9
**DDR1**	Receptor tyrosine kinase; potential treatment target.	10
**EGFR**	HIF induced; caveolin-1 regulated; established treatment target.	11, 12
**ENG**	Hypoxia regulated; potential treatment target.	13
**ITGA3**	Hypoxia regulated; potential treatment target.	14, 15
**ITGA5**	Hypoxia induced in colon cancer cells.	14, 16
**SLC16A3**	Part of pH-regulatory machinery; potential treatment target.	17, 18
**SLC2A1, SLC2A3**	HIF induced; major role in Warburg effect and hypoxic metabolism; associated with poor patient outcome; target of PET tumor imaging; potential treatment target.	19
**ALPP**	Overexpressed in several tumor types; potential treatment target.	20, 21
**IGFR1**	Hypoxia induced; potential treatment target.	22, 23
**IGFR2**	Mainly described as a tumour suppressor.	24
**ITGA1, ITGA2, ITGB1**	Widely implicated in tumor biology and as potential treatment targets; hypoxic induction of ITGB1.	14, 25, 26
**MET**	Widely implicated in tumor biology and as a potential treatment target; few reports on hypoxic regulation.	27, 28
**PODXL**	Associated with increased aggressiveness of various tumour types; potential surface marker of cancer cell stemness.	29
**ROR2**	HIF induced; potential treatment target.	30, 31

1) Robertson, N. et *al. Cancer Research* (2004) **2)** Pastorekova, S. et *al. BJU Int.* (2008) **3)** Carter, D., Lieber, A. *FEBS letters* (2014) **4)** Macor, P. et *al. Cancer res.* (2007) **5)** Ruf, M. et *al. Clin. Cancer. Res.* (2015) **6)** Law, C. L. *Cancer Res.* (2006) **7)** Guan, G. et *al. Cancer lett.* (2015). **8)** Brennecke, P. et *al. Clin. Exp. Metastasis.* (2014) **9)** Egorova, A. et *al. J. Gene. Med.* (2014) **10)** Kothiwale, S. *Drug Discov. Today.* (2015) **11)** Franovic, A. et *al. Proc. Natl. Acad. Sci. U. S. A.* (2007) **12)** Wang, Y. et *al. Proc. Natl. Acad. Sci. U. S. A* (2012). **13)** Debebe, Z., Rathmell, W.K. *Pharmacol. Ther.* (2015) **14)** Saller, M. et *al. Biochem. Biophys. Res. Commun.* (2012) **15)** Xiao, K. *Cancer res.* (2012) **16)** Koike, T. et *al. Proc. Natl. Acad. Sci. U. S. A.* (2004) **17)** Kim, Y. et *al. Hum. Pathol.* (2015) **18)** Marchiq, I. et *al. Cancer Res.* (2015) **19)** Vaupel, P. et *al. Cancer Metastasis Rev.* (2007) **20)** Dua, P. et *al. Cancer Res.* (2013) **21)** Dua, P. et *al. Nucleic Acid Ther.* (2015) **22)** Murakami, A. et *al. PloS one* (2014) **23)** Olmos, D. et *al. Lancet Oncol.* (2010) **24)** Martin-Kleiner, I. et *al. Cancer Lett.* (2010) **25)** Finger, E.C. et *al. Cancer Metastasis Rev.* (2010) **26)** Keely, S. et *al. FASEB J.* (2009) **27)** Pennacchietti, S. et *al. Cancer Cell.* (2003) **28)** Furlan, A. et *al. Cancer Res.* (2014) **29)** He, J. et *al. J. Proteome Res.* (2010) **30)** Wright, T.M., Rathmell, W. K. *J. Biol. Chem.* (2010).
